# Determination of normal post-mortem computed tomography (PMCT) parameters for the temporomandibular joint

**DOI:** 10.1007/s00414-025-03512-3

**Published:** 2025-05-22

**Authors:** Megane Beaugeois, Chris O’Donnell, Lyndal Bugeja, Richard Huggins, Joanna Glengarry

**Affiliations:** 1 Victorian Institute of Forensic Medicine (Forensic Services), 65 Kavanagh Street, Southbank, Victoria Australia; 2 Department of Forensic Medicine, Monash University, 65 Kavanagh Street, Southbank, Victoria Australia; 3 Oral and Maxillofacial Surgery Department, Austin Health, 145 Studley Road, Heidelberg, Victoria Australia

**Keywords:** Post-mortem, Post-mortem computed tomography (PMCT), Temporomandibular joint (TMJ), Temporomandibular joint dislocation, Radiology, Medicolegal death investigation, Forensic radiology

## Abstract

Post-mortem radiology, particularly post-mortem computed tomography (PMCT), has become an important tool in forensic death investigation, offering valuable insights into the cause and manner of death. However, the interpretation of post-mortem findings requires understanding the normal anatomical changes that occur after death, as artifacts can mimic pathology, complicating diagnosis. The objective of this study was to define the normal configuration of the temporomandibular joint (TMJ) in the post-mortem setting, using a newly developed measurement method, the TMJ PAT (perpendicular assessment tool), to assess the relationship between the mandibular condyle and the articular eminence on PMCT scans. A retrospective analysis was conducted on 100 non-decomposed, non-traumatic adult deaths. The TMJ configuration was assessed bilaterally using PMCT scans, and the condylar position was measured in relation to the articular eminence. Results indicated that in 99% of cases the apex of the condyle was positioned at or posterior to the eminence. The study established that a positive TMJ PAT measurement, indicating an anteriorly displaced condyle, is abnormal and suggests a potential pathology, such as temporomandibular joint dislocation (TMJD). Interrater reliability of the method was strong, demonstrating its potential utility in forensic pathology practice. This method can be applied by those with limited expertise in forensic radiology, making it valuable for use in diverse settings, including resource-poor environments. The study’s findings enhance the understanding of post-mortem TMJ anatomy and provide a reliable tool for distinguishing normal from abnormal TMJ configurations, crucial for accurate analysis in the death investigation setting. Future research should explore the application of this method in cases involving trauma and hanging, to further investigate phenomena such as Suspension-Associated Dislocation of the TMJ (SAD TMJ).

## Introduction

Modern medicolegal death investigation provides the forensic pathologist with a range of tools in the diagnostic armamentarium, one of which is the use of post-mortem (PM) radiology, in the form of computed tomography (CT) or magnetic resonance imaging (MRI). PMCT is a permanent digital record of the deceased and has utility in the determination of cause of death and documentation of natural disease and trauma [1]. It is best utilized as an adjunct to autopsy, and the two investigative modalities are complementary and are powerful when used together in experienced centers [1]. It has become apparent that PM radiology is not the same as clinical radiology, with so-called artifacts of death causing potential confusion with pathological processes [2]. Mimics of natural disease may be seen on imaging of the brain [3, 4] and lungs [5] for example. What might be diagnosable as trauma in the living, may in fact be an artifact of PM position [6]. Post-mortem decompositional changes may trap the unwary into calling a finding significant, when it is in fact merely artifact [7]. As such, it is essential that pathologists and radiologists are cognizant of such pitfalls.

The forensic pathologist’s role is not just to provide a cause of death, but to assist the relevant authority in their determination of the manner of death through the provision of medical evidence to aid in reconstructing the circumstances of death. In cases of death due to injury, one needs to consider not just the injuries that may be expected in the circumstances, but also unexpected injuries that may counter the scenario. The proviso is that one should not misinterpret artifacts as true injury, leading to unnecessary investigation and unwarranted anxiety for the next of kin.

We have recently described two cases of temporomandibular joint (TMJ) dislocation in association with deaths arising from self-inflicted hanging [8]– a previously unrecognized phenomenon in the literature. This finding is hypothesized to represent an artifact of ligature suspension (the entity of Suspension-Associated Dislocation of the TMJ [SAD TMJ]), however other causal possibilities exist that require exploration including antemortem or perimortem trauma, and decomposition. Trauma was excluded in both of our index cases. It is well-recognized that as decomposition progresses, disarticulation of joints occurs due to the breakdown of soft tissues, so dislocation of the TMJ may therefore be a normal finding in cases with advanced decomposition. However, our two index cases were fresh and were without putrefactive changes, making decomposition an unlikely explanation.

To investigate this issue further, it is clear that one needs to determine what constitutes a normal TMJ in the post-mortem setting. To our knowledge, this is not well established as there is currently no scientific literature that defines a normal or abnormal TMJ configuration in the post-mortem setting, with literature focusing on pathological changes in the TMJ [9, 10], or the findings of normal antemortem TMJs [11]. Clinically, a TMJ dislocation (TMJD) is defined as one in which the mandibular condyle is positioned anteriorly to the articular eminence with the jaw being unable to return to a closed position without the requirement of a specific maneuver by the patient or a clinician [12]. It is also normal in life for the condyle to move anterior to the eminence during wide mouth opening. Clinical criteria cannot be applied to decedents as a complaint of being unable to close the jaw cannot be assessed, and assessment of jaw movement is limited by post-mortem factors such as rigor mortis and decomposition [13, 14], rather than anatomy. Additionally, no scientific literature exists to describe how one might assess the position of the condyle on PMCT scans. This study aimed to address these gaps in knowledge by firstly defining the constitution of normal TMJ configuration (NorTMJCon) in the post-mortem setting, then establishing PMCT parameters to develop a reliable method of measuring the position of the condyle on PMCT scans for the purpose of distinguishing between normal and abnormal TMJ configurations (AbTMJCon) in order to provide reproducible parameters on which to determine the presence or absence of TMJD.

## Method

A retrospective cross-sectional study design was applied to determine the normal configuration and alignment of the TMJ in the post-mortem setting among adult cases of non-traumatic, non-decomposed deaths reported to the coroner in our jurisdiction, which is a major urban center with a population of over 6 million people. Deaths which appear to be unnatural or unexpected are notified to the coroner and approximately 7,000 deaths are investigated each year [15].

Participant data comprised 100 deaths notified to the coroner selected consecutively from January 1st, 2023, that met the following eligibility criteria: (i) the decedent was an adult aged between 18 and 80 years; (ii) the cause of death was non-traumatic; (iii) the decedent’s body was not decomposed; (iv) a PMCT scan was performed and the image was accessible within the imaging software; (v) an autopsy and/or external examination was performed and the post-mortem examination report was available for review. Participants were excluded if: (i) the decedent had a known TMJ abnormality/injury in life; (ii) the cause of death was traumatic (as trauma, such as blows to the jaw, can cause TMJD) [16]; (iii) the body was decomposed (as the process of decomposition causes tissue breakdown resulting in disarticulation of joints including the TMJ) [14]. Additional variables included: (i) case administration (study case number; institute case number; year); (ii) demographic and case characteristics (age; sex; height; weight; BMI; decomposition of head and neck; cause of death; manner of death; suspicious circumstances); (iii) method of examination (autopsy; PMCT; additional examinations); (iv) TMJ specific data (mouth position; apparent TMJ displacement; condyle in relation fossa [right and left]). Because interrater reliability testing is recommended for heterogeneous samples greater than 30 [17] a sample size of 100 cases was chosen to increase the robustness of the interrater reliability analysis. Participants were assessed for decomposition in accordance with the decomposition scoring method established by Megyesi et al. [18]. Post-mortem photography was used to determine stages of decomposition. As different body regions can undergo decomposition at different rates [14], only the head and neck regions were assessed. Participants categorized as ‘fresh’ were included, and participants categorized as decomposed (early, advanced, skeletonization) were excluded.

A method of measuring the distance of the mandibular condyle from the articular eminence via PMCT scans was devised by a forensic radiologist and forensic pathologist using the assisted perpendicular tool (rephrased and shortened to ‘PAT’ for ease of use) provided by the imaging software, syngo.via (version VB60A software, Siemens Healthineers, Erlangen, Germany). CT technique at our institution includes a head-to-neck scan range, at 120 kVp, 420 effective mAs, 0.5 mm slice thickness, pitch of 0.6, rotation time of 0.55 s, 500 mm field of view, and reconstruction kernel of Hr68h. Data extraction was then performed independently by three researchers: an honors student who received training from a forensic radiologist over a 1-day period to learn the technique; a forensic pathologist with over a decade of PMCT experience; a highly experienced forensic radiologist. Measurements performed by the forensic radiologist were used as the gold standard.

### The TMJ PAT measurement method

**Fig. 1:**
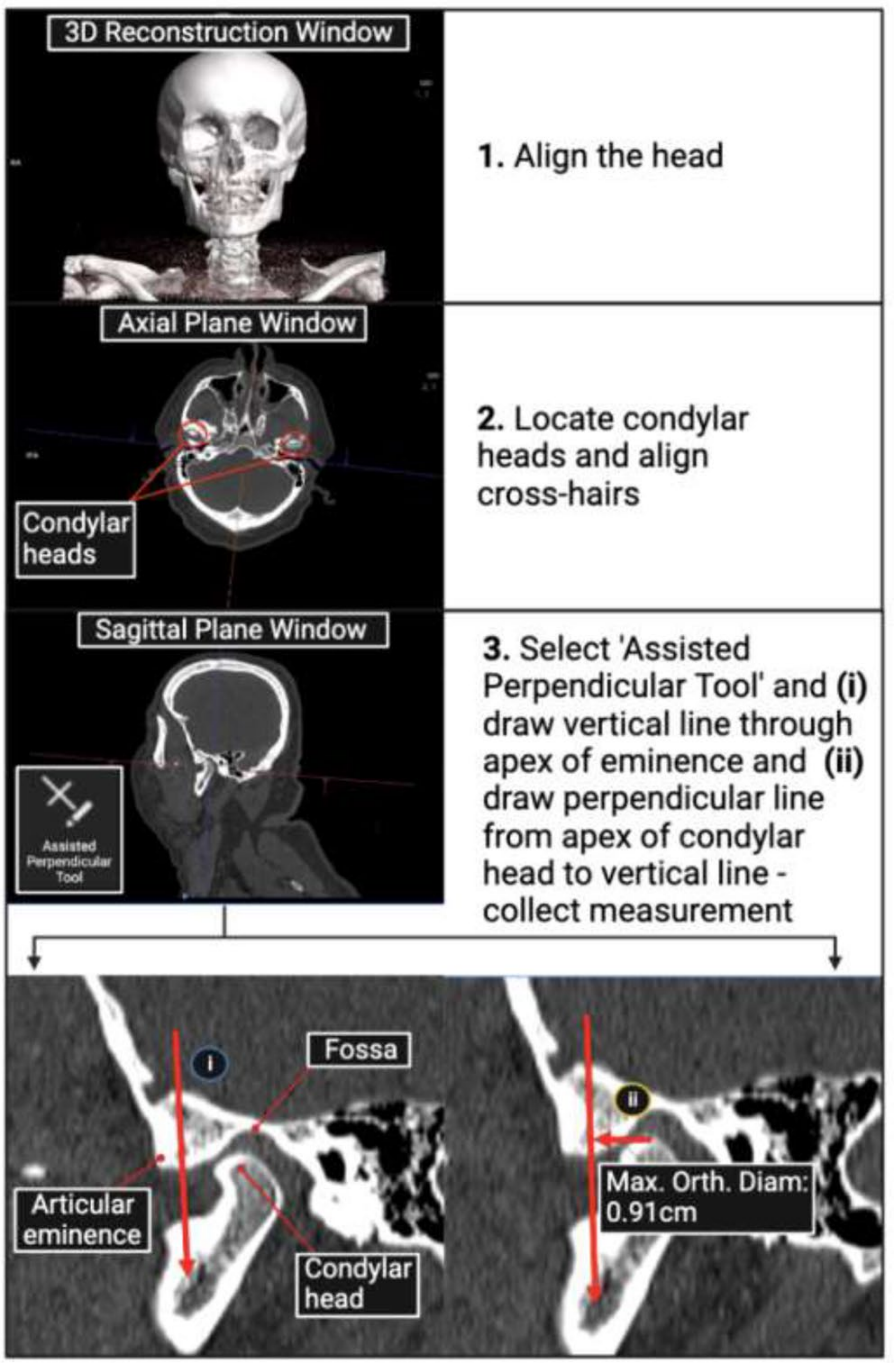
Flowchart of the temporomandibular joint (TMJ) PAT (perpendicular assisted tool) measurement method devised by the research team using post-mortem computed tomography (PMCT) scans using the imaging software, syngo.via. The three-dimensional (3D) reconstructions were used to determine whether abnormal TMJ configurations could be accurately identified visually

The TMJ PAT measurement method is described in Fig. 1. The three-dimensional (3D) reconstruction of the PMCT scan of the head and neck was firstly assessed using the 3D reconstruction window in syngo.via. Here, it was determined whether any TMJ abnormalities or pathologies could be identified, whether the TMJ appeared dislocated upon visual inspection, and the mouth position (open/partially open/closed) of the participant. Post-mortem changes, such as rigor mortis, make it difficult in some instances for the deceased person to be positioned in a straight, forward-facing position on the gantry table, as would regularly be required during clinical imaging procedures. The head of the participant was therefore aligned using the post-processing software (syngo.via) such that it was positioned in a normal anatomical orientation (forward-facing with the outer canthus of the orbits in a horizontal line with the external auditory meatus). Next, the mandibular condylar heads were located in the axial plane, and the multiplanar reconstruction crosshairs were centered over the right condyle so that it was sectioned equally into axial, sagittal and coronal planes. The sagittal plane window was then expanded and visual assessment was made to determine whether the condyle appeared to be positioned in or out of the mandibular fossa. The PAT tool was then selected and a vertical line was drawn through the apex of the eminence, followed by a perpendicular line from the apex of the condyle to the first vertical line. The ‘maximal orthogonal diameter’ measurement output was recorded, representing the right ‘TMJ PAT measurement’.

**Fig. 2:**
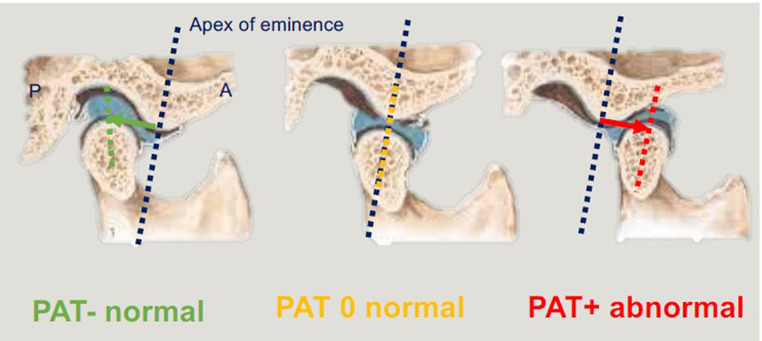
If the condyle is behind the eminence, in the fossa where it should be (green), this is a PAT negative measurement and is normal. If the condyle is at the eminence (yellow), this is a PAT zero measurement, which is normal. However, if the condyle is anterior to the eminence (red), this is PAT positive, which is abnormal and needs explanation

For the purpose of signifying the condyle’s position in relation to the articular eminence, the TMJ PAT measurement was assigned a ‘negative’ or ‘positive’ sign if the condyle was positioned posterior or anterior to the eminence, respectively. If the condyle was positioned directly over the eminence, the TMJ PAT measurement was zero (Fig. 2). The aforementioned steps were then repeated to measure the left TMJ. The TMJ configuration of each participant was determined using the right and left TMJ PAT measurements. A participant was deemed to have a NorTMJCon if a negative or zero TMJ PAT measurement was obtained bilaterally, or an AbTMJCon (i.e. TMJD) if a positive TMJ PAT measurement was obtained unilaterally or bilaterally.

Established clinical criteria for the assessment of TMJD were used to form our rationale as to why a TMJ PAT measurement of zero was classified as being within the limits of a NorTMJCon in this study. Clinically, a wide-open mouth position (which may occur while yawning for example) can cause the condyle to be temporarily positioned anteriorly to the eminence. This is deemed a normal finding if the patient can close their mouth again and is not, in itself, a predictor of TMJD but demonstrates the range of TMJ hypermobility in the population. The diagnosis of TMJD is made if the mouth is unable to return to a closed position without the patient or a clinician performing a specific manipulative maneuver [12]. It is not possible to determine if a decedent can close their mouth as (a) it is not possible to assess a complaint of being unable to close the mouth and (b) assessment of jaw movement is limited by post-mortem factors such as rigor mortis (which may render the jaw immovable) and decomposition (which may cause apparent TMJ hypermobility due to muscle and ligamentous breakdown), so it could not be definitively established whether a zero TMJ PAT measurement was within the normal range of TMJ hypermobility or indicative of TMJD. A conservative approach was therefore taken to classify a zero TMJ PAT measurement as being within the range of a normal post-mortem TMJ configuration to avoid over-reporting the numbers of TMJD in the results.

Data cleaning was performed using Microsoft Excel, then data was imported into the statistical software, GraphPad Prism to perform normality testing using the Kolmogorov-Smirnov test and descriptive statistics. The Kolmogorov-Smirnov test was selected as it is the recommended normality test for larger samples (*n* > 50) [19]. TMJ PAT measurements were imported into Statistical Package for Social Sciences (SPSS) to perform inter-rater reliability tests. The test was repeated three times using the following data sets taken by three raters: (a) right TMJ PAT measurements; (b) left TMJ PAT measurements; (c) combined right and left TMJ PAT measurements. The test was then repeated three times using the following data sets taken by two raters: (a) right TMJ PAT measurements; (b) left TMJ PAT measurements; (c) combined right and left PAT measurements. The purpose of performing repeated tests was firstly to determine the reliability between measures taken by an experienced forensic radiologist, a forensic pathologist with experience in analyzing PMCT scans and a novice in the field, then to assess the reliability between measures taken by an experienced forensic radiologist and a novice in the field. The interrater reliability test is recommended for testing measurements performed by 3 or more raters [17] therefore the results of the test applied to two raters must be interpreted with caution.

## Results

Among the 100 deaths, 62/100 (62.0%) were male and 38/100 (38.0%) were female. The median age, height, weight and BMI among these deaths was 64 years (IQR = 51–78 years, range = 70 years), 166 cm (IQR = 161–172 cm, range = 50 cm), 76 kg (IQR = 60–90 kg, range = 188 kg) and 26 kg/m^2^ (IQR = 23–32 kg/m^2^, range = 68 kg/m^2^), respectively. The cause of death was determined to be natural in 78/100 (78.0%). In all deaths, the circumstances were deemed to be ‘non-suspicious’ (348/348, 100.0%). In 61/100 (61.0%) deaths the post-mortem procedure consisted of an external examination only, and additional examinations (including toxicology tests) were performed for all deaths.

The mouth position was determined to be closed in 50/100 (50.0%) deaths and TMJ pathology was present in 8/100 (8.0%) deaths, comprising degenerative changes. From the review of the 3D reconstruction of the PMCT scans, TMJ displacement seemed *apparent* in 16/100 (16.0%) deaths. Assessment of the condylar head in relation to the mandibular fossa on the sagittal window planes of the PMCT scans determined that the right condyle *appeared* to be out of the fossa in 6/100 (6.0%) deaths and the left condyle *appeared* to be out of the fossa in 11/100 (11.0%) deaths. A summary of results is listed in Table 1.

**Table 1:** Sociodemographic, incident factors, method of examination and TMJ specific data

			Participants (*n* = 100)	
			n	%
Sociodemographic characteristics	Sex	Male	62	62.0
		Female	38	38.0
	*Age* (years): median (IQR), range		64 (51–78), 70	
	*Height* (cm): median (IQR), range		166 (161–172), 50	
	*Weight* (kg): median (IQR), range		76 (60–90), 188	
	*BMI* (kg/m^2^): median (IQR), range		26 (23–32), 68	
Incident factors	Manner of death	Natural	84	84.0
		Unintentional	14	14.0
		Suicide	2	2.0
	Cause of death	Natural	78	78.0
		External: drugs/alcohol	14	14.0
		External: post-operative complications	5	5.0
		Combined: drugs and alcohol	2	2.0
		External: choking on food	1	1.0
	Suspicious circumstances	Yes	0	0.0
		No	100	100.0
Method of examination	Autopsy performed*	Yes	39	39.0
		No	61	61.0
	Additional examinations performed, including toxicology	Yes	100	100.0
		No	0	0.0
TMJ specific data	Mouth position	Closed	50	50.0
		Partially open	40	40.0
		Wide open	10	10.0
	TMJ pathology	Present	8	8.0
		Absent	92	92.0
	Apparent TMJ displacement upon view of 3D reconstruction of PMCT	Yes	16	16.0
		No	84	84.0
	Appearance of right condylar head in relation to fossa on sagittal plane view of PMCT	In fossa	94	94.0
		Out of fossa	6	6.0
	Appearance of left condylar head in relation to fossa on sagittal plane view of PMCT	In fossa	89	89.0
		Out of fossa	11	11.0

Abbreviations: TMJ, temporomandibular joint, 3D, three-dimensional, PMCT, post-mortem CT scan, n, sample number, IQR, interquartile range

*Autopsies were performed in addition to external examinations. Participants who did not receive an autopsy underwent external examinations only

### The configuration of a normal TMJ after death

Of the total TMJ PAT measurements performed, no positive PAT measurements (AbTMJCon) were identified. Negative bilateral TMJ PAT measurements were identified in 99/100 (99.0%) deaths and the TMJ PAT measurement in one (1.0%) death was determined to be zero bilaterally (NorTMJCon). A summary of the measurements performed by the three raters are shown in Table 2. The mean of the total combined right and left TMJ PAT measurements among deaths taken by the three raters was − 1.00 cm (SD = 0.29 cm). Calculations of the mean TMJ PAT measurement ± 3 standard deviations are outlined in Table 3. The mean TMJ PAT measurement ± 3 standard deviations were − 0.13 cm and − 1.87 cm.

**Table 2:** TMJ PAT measurement results

TMJ PAT measurements	Rater	Total *n* measurements	Median (cm)	IQR (cm)	Range (cm)	Mean (cm)	SD (cm)
Right TMJ	1	100	-1.01	-1.20 - (-0.86)	1.43	-1.01	0.25
	2	100	-1.02	-1.28 - (-0.85)	1.96	-1.10	0.36
	3	100	-1.05	-1.17 - (-0.88)	1.49	-1.03	0.25
	1,2,3	300	-1.02	-1.22 - (-0.86)	2.04	-1.05	0.29
	2,3	200	-1.03	-1.18 - (-0.86)	1.54	-1.02	0.25
Left TMJ	1	100	-1.00	-1.18 - (-0.78)	1.48	-0.97	0.29
	2	100	-0.94	-1.04 - (-0.79)	1.24	-0.89	0.26
	3	100	-1.03	-1.17 - (-0.89)	1.42	-0.99	0.27
	1,2,3	300	-0.99	-1.14 - (-0.81)	1.59	-0.95	0.28
	2,3	200	-1.02	-1.17 - (-0.82)	1.59	-0.98	0.28
Right and left TMJ	1	200	-1.01	-1.18 - (-0.83)	1.48	-0.99	0.27
	2	200	-0.98	-1.15 - (-0.81)	1.96	-1.00	0.33
	3	200	-1.04	-1.17 - (-0.88)	1.49	-1.01	0.26
	1,2,3	600	-1.00	-1.16 - (-0.84)	2.04	-1.00	0.29
	2,3	400	-1.02	-1.17 - (-0.86)	1.59	-1.00	0.27

Abbreviations: TMJ, temporomandibular joint, PAT, perpendicular assisted tool, n, sample number, IQR, interquartile range, SD, standard deviation

Right and left TMJ PAT measurements of participants (*n* = 100) were performed independently by (1) honors student, (2) forensic pathologist and (3) forensic radiologist using the PAT measurement method devised by the research team

**Table 3:** Mean of a normal TMJ configuration TMJ PAT measurement ± 3 standard deviations

SD	Mean (-1 cm) + SD (cm)	Mean (-1 cm) - SD (cm)
± 1 SD (0.29 cm)	-0.71	-1.29
± 2 SD (0.58 cm)	-0.42	-1.58
± 3 SD (0.87 cm)	-0.13	-1.87

Abbreviations: TMJ, temporomandibular joint, PAT, perpendicular assisted tool, SD, standard deviation

The mean measurement of a normal post-mortem TMJ (-1 cm) was calculated by combining the right and left TMJ perpendicular assisted tool measurements of participants (*n* = 100) performed by three raters

### TMJ PAT measurement interrater reliability analysis

The results of the interrater reliability analyses are described in Table 4. Results showed that the total combined right and left TMJ PAT measurements taken by the three raters (MB, JG, COD) were of ‘good’ reliability based on the confidence intervals (ₖ = 0.809, CI = 0.759–0.851). Further, calculation of data reliability determined that 65.4% of the data was reliable which signifies a ‘strong’ level of agreement. Analysis of the total combined right and left TMJ PAT measurements taken by two raters (MB, COD) were of ‘excellent’ reliability based on the confidence intervals (ₖ = 0.924, CI = 0.900-0.943). Further, calculation of data reliability determined that 85.4% of data was reliable which signifies an ‘almost perfect’ level of agreement.

**Table 4:** TMJ PAT measurement interrater reliability test results

Assessment of measurements between raters	TMJ PAT measurements tested	Kappa (95% CI)	Level of reliability (based on 95% CI)*	% of reliable data**	Level of agreement (based on % of reliable data)***
1,2,3	Right TMJ	0.698 (0.579–0.788)	Moderate - good	48.7%	Moderate
	Left TMJ	0.912 (0.877–0.938)	Good - excellent	83.2%	Almost Perfect
	Right and left TMJ	0.809 (0.759–0.851)	Good	65.4%	Strong
2,3	Right TMJ	0.915 (0.873–0.943)	Good - excellent	83.7%	Almost Perfect
	Left TMJ	0.931 (0.898–0.954)	Good - excellent	86.7%	Almost Perfect
	Right and left TMJ	0.924 (0.900-0.943)	Excellent	85.4%	Almost Perfect

Abbreviations: TMJ, temporomandibular joint, PAT, perpendicular assisted tool, CI, confidence intervals,

Interrater reliability tested for right TMJ, left TMJ, and right and left TMJ PAT measurements of participants (*n* = 100) performed by all raters (honors student, forensic pathologist and forensic radiologist), as well as the least experienced rater with the “gold standard” (forensic radiologist)

*Level of reliability assessed in accordance with Koo and Li (2016) [17]

**Percentage of reliable data calculated by squaring Kappa

***Level of agreement assessed in accordance with McHugh (2012) [23]

## Discussion

To ascertain what is abnormal, one must understand what is normal. Forensic pathologists are cognizant of the fact that appearances and measurements after death are not necessarily the same as those during life [20, 21]. We therefore aimed to establish what constitutes a normal TMJ in the post-mortem setting by devising an objective, reliable, and accurate measurement tool that can be applied to PMCT imaging. This means one can thus detect whether the TMJ configuration is abnormal and allows consideration of the cause and whether this may be artefactual (for example, due to decomposition), or a real phenomenon (such as trauma or disease).

Application of the TMJ PAT measurement method devised by the research team to cases where no TMJ abnormality would be expected identified normal findings in all cases, that is, negative or zero TMJ PAT measurements in 99.0% and 1%, respectively. A normal mandibular condyle was found to be 1.00 cm behind the articular eminence, with three standard deviations still giving a negative PAT measurement (mean of the total TMJ PAT measurements − 1.00 cm ± 3 SD = -0.13-(-1.87) cm). Thus, we propose that a normal post-mortem TMJ configuration is defined as one in which the apex of the condyle is at or posterior to the apex of the articular eminence (i.e. a zero or negative TMJ PAT measurement).

Our study demonstrated that visually inspecting the 3D reconstructions of PMCT scans is not a reliable way of identifying TMJD. When assessing the 3D reconstructed views, TMJ displacement appeared apparent in 16.0% of cases, but when measurements were taken on these cases using the sagittal bone window, there were in fact no positive TMJ PAT measurements (no AbTMJCon), indicating the appearances on the 3D reconstruction may be misleading in suggesting the presence of TMJD when it is not actually present.

The PAT measurement method was developed to use standard CT viewing software to determine the relationship of the mandibular condyle to the articular eminence, creating an objective way of assessing the TMJ. Based on the results of the interrater reliability tests, there was a ‘strong’ level of agreement between measurements performed by a forensic radiologist, a forensic pathologist experienced in viewing post-mortem imaging and a novice in the field. Analysis of measurements performed by a forensic radiologist and the novice showed an ‘almost perfect’ level of agreement, although results must be interpreted with caution as the interrater reliability test is not recommended for testing on less than three raters [17]. Our results demonstrate that the TMJ PAT measurement method appears to be a reliable test and thus, its application in the post-mortem setting will be of use. In addition, it was shown that the test could be taught to and performed by an individual with no prior experience in analyzing the TMJ on PMCT scans.

### Limitations

The measurements were only performed and validated among adults, so the PAT cannot be applied to pediatric cases. Information sources available to the researchers included police reports, family, and medical records. These generally lacked information about the deceased’s TMJ history (such as previous TMJD), so information about a propensity for jaw dislocation may have been under-reported.

While none of the cases were considered “suspicious” after a full police and coronial investigation, the possibility of unwitnessed or occult trauma as a cause of TMJD was minimized but could not be entirely excluded.

We assessed the level of decomposition using a method developed for assessment of the external observable features of the deceased, but those reviewing cases purely radiologically may not have access to such information, and we suggest that the radiological alteration index of Egger et al. [22] would be applicable in this situation.

### Future Research

Now that normal and abnormal post-mortem TMJs have been defined and a reliable measurement tool developed, it needs to be applied to larger populations, including those with potentially abnormal TMJ configurations, such as deaths due to hanging allowing “real-world” application of our method, how it may inform the manner of death, and exploration of the purported entity of Suspension-Associated Dislocation of the TMJ (SAD TMJ).

### Conclusion

In summary, we have established a robust method for measuring the TMJ configuration in a post-mortem setting and therefore being able to distinguish a normal from an abnormal TMJ. This method can be applied by a novice in the use of PMCT scans and is accessible to those without expert forensic radiology expertise and support available and thus can be used in resource-poor settings.

### Take home points

1. Medicolegal death investigation involves the identification and determination of the significance of natural disease, trauma and other abnormalities. To do this, one needs to know what constitutes “normal” in the post-mortem setting.
2. We have devised a method for measuring the position of the TMJ after death and determined normal values using PMCT imaging.
3. The mandibular condyle is normally located at or behind the articular eminence after death. Location of the condyle anterior to the eminence is abnormal and indicates an abnormal TMJ configuration, where consideration should be given to the cause, with reference to the circumstances of death.
